# Tokophobia and Anxiety in Pregnant Women during the SARS-CoV-2 Pandemic in Poland—A Prospective Cross-Sectional Study

**DOI:** 10.3390/ijerph19020714

**Published:** 2022-01-09

**Authors:** Marta Makara-Studzińska, Kornelia Zaręba, Natalia Kawa, Dorota Matuszyk

**Affiliations:** 1 Department of Health Psychology, Institute of Nursing and Midwifery, Jagiellonian University Medical College, 31-501 Cracow, Poland; marta.makara-studzinska@uj.edu.pl (M.M.-S.); instpiel@cm-uj.krakow.pl (N.K.); 2 First Department of Obstetrics and Gynecology, Center of Postgraduate Medical Education, 01-004 Warsaw, Poland; 3 Laboratory of Fundamentals in Obstetric Care, Institute of Nursing and Midwifery, Faculty of Health Sciences, Jagiellonian University Medical College, 58 Zamoyskiego St., 31-523 Cracow, Poland; dorota.matuszyk@uj.edu.pl

**Keywords:** tokophobia, perinatal anxiety, SARS-CoV-2, perinatal depression, perinatal stress

## Abstract

SARS-CoV-2 coronavirus emerged in the world at the end of 2019. The introduction of a number of restrictions had a significant effect on numerous aspects of human life with particular influence being exerted on pregnant women and their sense of security. The study aimed to assess the level of anxiety and its main determinants in women in the third trimester of pregnancy during the coronavirus pandemic. The study technique included the present purposely designed questionnaire, Labor Anxiety Questionnaire (KLPII), and the State-Trait Anxiety Inventory (STAI). The study was conducted in a group of 315 women in the third trimester of pregnancy. A total of 258 women (81.9%) completed the questionnaire in May 2020, and 57 of them (18.1%) completed it in October 2020. The overall analysis of the Labor Anxiety Questionnaire and the STAI inventory revealed a high level of anxiety, particularly situational anxiety, in pregnant women during the SARS-CoV-2 pandemic. The age and financial status of the women were the factors which contributed to the intensification of tokophobia. Women interviewed in October 2020 were characterized by higher tokophobia levels compared to the respondents included in May 2020. It seems justified to in-crease the vigilance in the diagnostics of possible mental disorders in the perinatal period during pandemic.

## 1. Introduction

Tokophobia has been studied by doctors, psychologists and sociologists for 40 years. However, it has not been clearly defined so far [1,2]. The term “tokophobia” was first used by Hofberg and Brockington in 2000. They defined tokophobia as intense, pathological and unjustified anxiety which induces fear and, in some cases, makes women avoid labor with the simultaneous wish to have a child [3].

Tokophobia occurs in approx. 6–10% of pregnant women. The results of research conducted in Sweden in 2007 revealed that 80% of pregnant women might experience mild forms of fear related to labor, while 2% experienced extremely strong phobia-like fear of labor [4]. Polish research conducted in 2017 in women in the third trimester of pregnancy showed low or average levels of tokophobia in 65.66% of pregnant women, elevated levels in 18.18%, high in 10.10% and very high in 6.06% [1].

Tokophobia is a specific and multidimensional psychological phenomenon which is linked to the way of the cognitive interpretation of labor and is classified as an anxiety disorder [1,3,4]. Distinguishing between anxiety and fear is a problematic issue. Strong fear associated with labor was found to be correlated with a high predilection for developing anxiety [1]. Tokophobia is related to the fear of pain and injury with episiotomy being the most common cause, the loss of control and labor pain, and situations of threat to the life or health of the child [4]. Some authors indicated culture-related factors in the context of the incidence of tokophobia and the fact that the problem was more common in urban areas. According to available literature, the factors which predispose for the occurrence of tokophobia include: being young, low level of education, difficult financial situation, everyday stress, no support from one’s environment, solitude, a history of trauma, susceptibility to being hurt, neurotic personality, previous cesarean section, a disease in the previous child or the mother, the lack of suitable knowledge about reproduction [4]. The severity of tokophobia may also be affected by previous obstetric experiences, such as: miscarriage, ectopic pregnancy, stillbirth, premature delivery or teenage pregnancy, and experiencing a pregnancy for the first time [1]. Stress-inducing events during pregnancy are also of importance [5]. Makara-Studzińska reported that tokophobia was frequently experienced by women who were not married, unemployed and in a poor financial situation [6]. According to Sjögren, tokophobia was also associated with the lack of confidence in the medical personnel [7].

Pregnancy is a trigger of numerous positive and, frequently, also negative emotions [8,9,10]. It is also a period when new patterns of reaction are developed [11]. Emotional changes in pregnant women are influenced both by the personality, the current situation and environment of the woman. According to the literature, pregnant women are characterized by higher introversion, lower self-acceptance and lower sense of independence [10,12]. They are expressed as anxiety related to the perinatal period, labor in particular [13]. Low-intensity anxiety is experienced by about 80% of pregnant women, while severe anxiety is noted in 20% of them. The intensification of anxiety is observed in the third trimester of pregnancy with 71.5% of women manifesting highest levels of severe degree anxiety in Africa [14]. Anxiety is defined as unpleasant and unclear emotional status related to the anticipation of an external danger or originating from the inside of the body [6]. External stimuli triggering anxiety may include the media or opinions of others, while internal stimuli include one’s thoughts, concepts and memories [15]. Anxiety may be accompanied by the symptoms of mental, motor and autonomic arousal [16]. Pregnancy-related hormonal changes in the levels of cortisol, estradiol or prolactin may promote anxiety and depressed mood [6]. Anxiety-related reactions may also result from abnormalities in the network of connections between the prefrontal cortex with the amygdala [16].

The severity of tokophobia varies depending on the duration of pregnancy. The first trimester is the stage when intense changes occur in the woman’s body. Psychologically, it is a period of mental crisis or a stage of adaptation [10,12]. Women are usually then concerned about pregnancy loss. In 2019, research conducted by Lachowska and Szeliga in a group of pregnant women showed they were the most anxious about miscarriage, labor, hospitalization, and the life and health of the fetus [11]. The attention on the changes in the woman’s body is redirected to the fetus over time. During the second trimester the woman experiences numerous positive emotions, establishes a relationship with the fetus, feels its movements, shares the information of the changes with others and fantasizes about the fetus [10]. Negative emotions may be experienced in women in relation to the changing body, fear of intercourse or no acceptance of pregnancy [10,17]. Emotions during the last three months of pregnancy are closely associated with the approaching labor and it is undoubtedly the most difficult period. Pregnant women are afraid of labor pains and intrapartum complications, and feel incompetent as mothers [12,17]. Women also experience difficulties concentrating, remembering and performing intellectual work, which is associated with hormonal changes preparing women for labor.

SARS-CoV-2 coronavirus which causes COVID-19 appeared at the end of 2019. The coronavirus spread rapidly around continents which led to the outbreak of the pandemic [18]. The majority of economic sectors had been shut down until the third of May 2020 [18]. A total of 115,082,747 cases and 2,552,272 deaths had been confirmed worldwide until March 2 2021. In Poland, 1,719,708 persons suffered from and 44,008 died of COVID-19 [https://www.worldometers.info/coronavirus/, accessed on 2 March 2021]. The introduction of successive restrictions limiting interpersonal contact significantly influenced numerous aspects of human life. Gatherings were banned, the use of personal protective measures was recommended and getting around was only recommended if necessary. The pronounced majority of public venues were inaccessible [19]. Supposedly, the omnipresent slogan “stay at home” had never triggered such anxiety in the society. Limitations in the provision of health care significantly influenced prenatal care and delivery. Due to the lack of research results, at the beginning of the pandemic the Polish Gynecological Society recommended cesarean section as the mode of delivery in all women infected with SARS-CoV-2. The fear of becoming infected and the effects of lockdown measures, particularly changes in health care provision, had an enormous effect on perinatal women. Family birth was suspended and women were on their own in the hospital throughout the stay in the department the moment when the COVID-19 pandemic was announced. It was due to the fact that knowledge in this area was very limited in Poland and in many other countries. Women were also commonly separated from the neonate in order to prevent the transmission of the virus. The vision of being alone during labor, in a mask, with a constant concern about being infected in the hospital which would result in being quarantined were factors which intensified the already existing labor-related anxiety. The strict restrictions were not neutral for the psyche of pregnant women. Being alone during delivery is related to high levels of stress and anxiety, higher sensitivity to pain which has a negative influence on the physical and mental health of the mother, child, and the whole family.

Pregnancy is a physiological status when the immunity of the body is different due to the immune tolerance of developing fetus [20]. Some viral upper respiratory infections may have a more severe course in pregnant women than in other individuals. However, it has not been determined to date whether the course of COVID-19 differs in pregnant women compared to other people of similar age. According to the literature, premature delivery occurred in 41% of subjects, rupture of membranes was observed in 19% of women, while 14% developed preeclampsia. Moreover, one intrauterine death and one neonatal death were reported, 9% of neonates required intensive care and 43% cases had threatened fetal asphyxia. However, the authors of the meta-analysis emphasized that binding conclusions were difficult to formulate due to the small sample size and the retrospective nature of the research. Seemingly, some authors published results which showed that SARS-CoV-2 infection increased the percentage of cesarean sections. The underlying cause of such a correlation is unknown, but it may be associated with more common manifestations of threatened fetal asphyxia in mothers infected with SARS-CoV-2 [21].

Until recently, the presence of coronavirus has not been confirmed in breast milk. The most recent guidelines of the Royal College of Obstetricians and Gynecologists (RCOG) and WHO do not indicate the necessity to isolate an infected mother from the infant and to stop breastfeeding. Conversely, they recommend breastfeeding due to the fact that the neonate is provided with antibodies contained in breast milk [22].

Reports concerning new cases of infection and death, and the deteriorating situation in some countries had a significant effect on the intensification of psychological distress including restlessness, sadness, symptoms of depression and clinically significant anxiety [23]. Social isolation may constitute a trigger of mental disorders. The problems may be manifested as insomnia, depressed mood, anxiety, fear with somatization and anhedonia [18]. A crisis which occurred in a large group may exceed the resources of individuals necessary in dealing with stress [23]. Experiencing stressful events frequently leads to the assumption that one is incapable of coping with them. Each similar, difficult situation is viewed with increased anxiety or depressed mood which may result in the development of neurosis [24]. Researchers from Jagiellonian University analyzed the psychological reactions of people to the epidemic situation and demonstrated that, over time, anxiety may transform into belief in conspiracy theories claiming that the virus might have originated from a laboratory or that some groups aim at the depopulation of the planet [25]. Based on pre-pandemic publications which cover research on people being quarantined due to SARS and MERS, some groups may be listed as more susceptible to the psychological consequences of quarantine. The groups include individuals with mental disorders, children, adolescents, elderly people, minority groups, persons with lower socioeconomic status and women [26]. Individuals infected with coronavirus, quarantined or those who lost their jobs were the most exposed to severe stress [27]. Research conducted to date has shown that some groups manifested significantly higher levels of stress, anxiety and depression, i.e., women, students, patients with specific physical manifestations (e.g., muscle pain, dizziness), the elderly, persons with at least one concomitant disease, migrants, individuals with low levels of education and low complexity of professional tasks, those living with an elderly person [18]. The factors which alleviate the symptoms of anxiety and depression seem to include: being married, high financial status and higher levels of education [28]. The following factors were also viewed as protective ones: reliable, current and precise information concerning the epidemic (e.g., the accessibility of diagnostics and treatment, detailed local epidemic situation), information about the use of recommended preventive measures (e.g., hand hygiene, wearing a mask), reports concerning improvements of telemedicine techniques [18]. 

Anxiety experienced by pregnant women in relation to the COVID-19 pandemic exerts a considerable effect on the quality of life of young mothers, and the pre- and post-natal development of their offspring [10]. Therefore, the problem should be considered as significant and requiring detailed diagnostics. Hence, we decided to assess the level of anxiety and tokophobia in women in the third trimester of pregnancy and its main determinants in order to distinguish a group which is the most at risk of developing psychological complications and to introduce appropriate protective management.

## 2. Materials and Methods

### 2.1. The Aim of the Study

Detailed aims:

1. The assessment of the severity of tokophobia in the study group;
2. The assessment of state and trait anxiety in the study group;
3. The assessment of the influence of sociodemographic factors such as age, level of education, medical education, place of residence, location at which delivery took place, occupational activity and financial status on the severity of tokophobia and situational anxiety evaluated on the STAI scale;
4. The assessment of correlations between the duration of the pandemic and the level of tokophobia and state anxiety.

### 2.2. Research Hypotheses

1. The SARS-CoV-2 pandemic increases the level of anxiety in women in the 3rd trimester of pregnancy;
2. Sociodemographic factors influence the level of tokophobia in pregnant women;
3. The duration of the SARS-CoV-2 pandemic influences the level of tokophobia in women.

### 2.3. Material

The study was conducted with the diagnostic survey method involving the collection of data concerning the structural and functional attributes and the dynamics of social phenomena occurring in selected populations. Questionnaires were selected as the diagnostic method. Women were recruited randomly. The research was conducted via social media with the protection of personal data. Women were included into the study on the basis of their gestational age (third trimester) and the location when delivery took place (in Poland). The access to groups of pregnant women in social media was gained after we had sent enquiries to group administrators or accessed the groups automatically after completing a questionnaire concerning the aim of joining such a group. Our questionnaires were made available to pregnant women in 9 such groups. The study was conducted during two waves of the pandemic: in May 2020 and October 2020 via social media with the protection of personal data. The study was conducted during two time frames in order to assess whether the duration of the pandemic was a risk factor of tokophobia in pregnant women. The second group was smaller due to the limited access to patients during the second time frame related to sanitary restrictions. 

Inclusion criteria:

1. Consent to participate in the study;
2. Women in the 3rd trimester of pregnancy (27–41 weeks of gestation);
3. No psychiatric diseases.

Exclusion criteria:

1. No consent to participate in the study;
2. Women in the 1st and 2nd trimester of pregnancy;
3. Women treated for psychiatric diseases.

**Figure 1:**
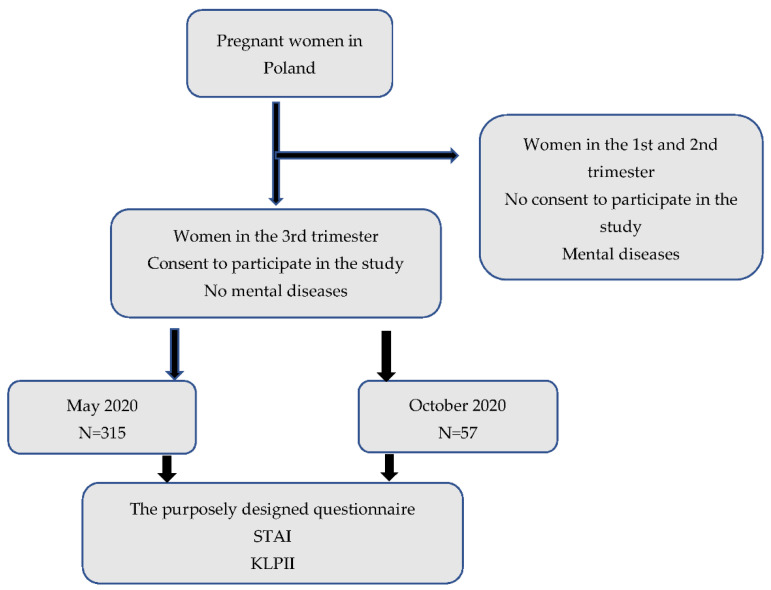
Study design.

After obtaining written informed consent the recruited patients were asked to complete anonymous questionnaires prepared for this study. Study design is presented in Figure 1.

The permission of the bioethical committee of Jagiellonian University Medical College (Collegium Medicum) was obtained (No. 1072.6120.65.). The study was conducted with the diagnostic survey method.

### 2.4. Method

The study technique included the present purposely designed Questionnaire, Labor Anxiety Questionnaire (KLPII), and the State-Trait Anxiety Inventory (STAI).

#### 2.4.1. The Purposely Designed Questionnaire

The purposely designed questionnaire was composed of 42 questions with 5 of them being open-ended. The preliminary questions of the questionnaire tackled the demographic and socioeconomic issues of the study group, i.e., age, level of education, place of residence, financial status and relationship status. As regards the criteria of study group selection it was important to ask about the month of gestation, date of completing the questionnaire, and the town in which delivery was planned. The questionnaire also included questions about the course of the present gestation, obstetric history, general medical history. Subsequent questions concerned issues significant from the viewpoint of the pandemic situation: having medical education, work from home, contact with infected people, anxiety regarding the present delivery and delivering in a mask. Questions also tackled the issue of the necessity, frequency and aims of leaving home during national quarantine, undergoing tests for the presence of coronavirus or anti-coronavirus antibodies. The remaining questions investigated the fear of becoming infected during hospitalization and fear of delivering in the absence of family or a friend. 

#### 2.4.2. Labor Anxiety Questionnaire (KLPII)

The Labor Anxiety Questionnaire (KLPII) revised version was authored by Paciorek and Putyński [29]. The Cronbach’s alpha reliability index calculated in the final assessment of 53 pregnant women was 0.69. In order to estimate the accuracy of KLPII a total of 53 women were assessed with the use of Labor Anxiety Questionnaire by Putyński (1997) with the correlation of r = 0.67 and Multi-component Anxiety Inventory by Daisy Schalling (MCA) with the obtained correlation with anxiety being r = 0.40. KLPII includes 9 questions formulated with the use of the Likert scale. Individual categories are assigned point values from 0 to 3 according to the following key: statements 2, 3, 5 values from 0 to 3, and statements 1, 4, 6, 7, 8, 9 values from 3 to 0. Therefore, the results may range from 0 to 27 points. The higher the score, the higher severity of tokophobia was observed. Basing on the mean and standard deviation (M = 11.98, SD = 2.14) obtained in validation group assessment the initial reference values for KLPII were determined as follows: from 0 to 13 points low and moderate level of tokophobia,

- From 14 to 15 points—elevated level of tokophobia;
- From 16 to 17 points—high level of tokophobia;
- From 18 to 27 points—very high level of tokophobia.

#### 2.4.3. State-Trait Anxiety Inventory STAI 

State-Trait Anxiety Inventory (STAI) is composed of two parts. In the parts investigating the level of situational anxiety trait anxiety the respondents selected one of the following values for their answers: 1, 2, 3, 4. Thus, the respondents could score from 20 to 80 points in each part. The higher the score, the higher state anxiety was observed.

The results of state anxiety and trait anxiety were divided into sten scores (sten 1–10). 

The sten scores of state anxiety and trait anxiety were divided into levels according to the following rule:

- From 1 to 3 sten scores—low state anxiety and trait anxiety;
- From 4 to 7 sten scores—moderate state anxiety and trait anxiety;
- From 8 to 10 sten scores—high state anxiety and trait anxiety.

The reliability of Japanese STAI Study for females was (state α = 0.02, trait α = 0.92), and Dutch version (α = 0.94) [30].

### 2.5. Statistics 

Statistical analysis was used to analyze the collected data. The significance of differences within individual groups was verified with the Chi2 test (χ^2^ Pearson) for such variables. Quantitative variables were presented with the use of the mean and standard deviation, the median, minimum and maximum. The Kolmogorov–Smirnov test was performed to compare the distribution of the analyzed variables with the normal distribution in order to select statistical methods. The correlations between quantitative variables within individual groups were tested with the one-way analysis of variance (ANOVA) for normally distributed variables. The Kruskal–Wallis ANOVA test was used to analyze correlations between quantitative variables which were not normally distributed within individual groups. Statistical significance was assumed at *p* < 0.05 for calculations in the present paper.

Analyses were conducted with IBM SPSS Statistics for Windows, Version 24.0., Armonk, NY, USA: IBM Corp. (Released 2016).

## 3. Results

### 3.1. Characteristics of the Group

The study was conducted in a group of 315 women in the third trimester of pregnancy. A total of 258 women (81.9%) completed the questionnaire in May 2020, and 57 (18.1%) respondents completed it in October 2020.

The average age of the respondents was 28 years (SD 4 years). Half of the respondents were at least 27 years old. The youngest patient was 17 and the oldest one was 39 years old. The majority of patients (72.4%) were married and over half of the respondents had higher education (58.4%) (Table 1). Medical education was declared by 32 respondents (10.2%). The majority of women assessed their financial status as good (57.1%) or very good (13.8%). Moreover, the majority of the respondents were not professionally active (66%). A total of 32 women (10.2%) worked from home.

Primiparas constituted 62.8% of the respondents and miscarriage was reported by 17.2% of women (Table 2). Almost all women attended regular gynecological consultations during the pregnancy (95.6%) (Table 2). Only one person (0.3%) underwent a blood test for the presence of anti-coronavirus antibodies and 314 women (99.7%) were not tested.

### 3.2. Anxiety Assessment

The severity of tokophobia was assessed by the respondents on the scale 0–5. The average score was 3 points (SD 1 point). However, the median was 4 points. Anxiety related to delivering in a mask was assessed by the patients at the average of 4 points (SD 1 pt) with the median of 5 pts. The average score concerning the fear of delivering without the presence of family or a friend was 4 points (SD 2 pts). The median was 5 points. The average score concerning anxiety related to the observance of procedures undertaken to prevent the spread of coronavirus by medical personnel was 3 points (SD 1 pt) with the median below 3.

The respondents were also asked about the frequency at which they checked COVID-19 statistics. A total of 12 respondents (3.8%) paid attention to the statistics every spare moment, 33 persons (10.5%) checked the statistics twice a day, 113 women (35.9%) did it once daily, 41 of them (13%) checked the incidence once or twice a week, and only 75 respondents (23.8%) paid no attention to the statistics.

#### 3.2.1. Labor Anxiety Questionnaire (KLPII)

The analysis of Labor Anxiety Questionnaire (KLPII) revealed that 80% of the respondents were afraid that the labor would last long, while only 38% had a feeling of controlling the situation. They were the most fearful as regards labor pains (88.3%). Unexpected complications were the cause of anxiety in 41% of the respondents. A total of 40% of the patients were worried about fetal injury during labor. Anxiety related to defects which might occur in the child were reported by 25.3%. Only 29.6% of the women were certain they would be calm and in control during labor, and 65.1% of the respondents were certain they would recover soon after delivery. A total of 60.9% of the respondents perceived waiting for labor as cheerful time.

The severity of anxiety was estimated in the study group basing on the results of the Labor Anxiety Questionnaire. The average severity of tokophobia was 16 points (SD 3 pts). Moreover, half of the respondents obtained no less than 16 points on this scale. The lowest score was nine, and the highest twenty-three points. Therefore, the overall analysis of the questionnaire revealed a high level of anxiety in the respondents. Only 50 respondents (15.9%) manifested low and moderate level of tokophobia, 78 persons (24.8%) were characterized by increased anxiety, 105 women (33.3%) had high levels of tokophobia and very high levels were noted in 82 respondents (26%). Statistical tests revealed significant differences in the levels of tokophobia. The majority of respondents were characterized by high (33.3%) or very high (26.0%) levels of tokophobia (Table 3).

#### 3.2.2. State-Trait Anxiety Inventory (STAI)

The mean value of situational anxiety based on the State-Trait Anxiety Inventory (STAI) was 46 points (SD14 points). Half of the respondents obtained no less than 47 points on this scale. The sten results were: mean—7 sten scores (SD 3 sten scores). Half of the women obtained no less than 8 sten scores. Low state anxiety was noted in 10.8% of the respondents (1–3 sten scores), 117 persons were characterized by moderate state anxiety (4–7 sten scores) and in 52.1% state anxiety was high (8–10 sten scores).

Statistical tests performed to assess differences in state anxiety revealed significant differences in the levels of situational anxiety, i.e., the majority of the respondents were characterized by high levels of situational anxiety (52.1%) (Table 4).

Statistical analyses revealed that the level of education including medical education, the place of residence, professional activity and the location of delivery had no influence on the level of tokophobia. However, the age of the respondents was found to influence the severity of tokophobia assessed basing on the present authors’ questionnaire. Older women were characterized by lower tokophobia levels than younger ones (Table 5). The analysis of variance (ANOVA) of state and trait anxiety revealed that age had no effect on state anxiety (H test 0.347, *p* = 0.841).

Statistical analyses revealed that the level of education including medical education, the place of residence, professional activity and the location of delivery had no influence on the level of tokophobia. However, financial status was found to have a significant effect on state anxiety. Women with average financial status were characterized by higher state anxiety than women with good or very good status. Moreover, persons with poor and very poor financial status presented high state anxiety (Table 6).

Moreover, a statistically significant correlation was shown between the time during the wave of the pandemic and the severity of anxiety. The respondents who completed the questionnaire in October 2020 were characterized by higher levels of anxiety than those completing it in May 2020 (Table 7). Additionally, a statistically significant correlation was confirmed between the time during the wave of the pandemic and the level of situational anxiety. The pronounced majority of the respondents interviewed in October 2020 presented high state anxiety, while women interviewed in May 2020 presented moderate or high state anxiety (Table 8).

## 4. Discussion

The overall analysis of Labor Anxiety Questionnaire and the STAI inventory revealed a high level of anxiety in pregnant women during the SARS-CoV-2 pandemic. The state of persistent anxiety experienced prior to specific situations is mostly associated with the avoidance of its causes. Therefore, more and more women (14% worldwide) decide to deliver via cesarean section [31]. In extreme cases, tokophobia may be associated with no wish to start a family and, as a consequence, having no children [32]. However, young mothers are unable to avoid the SARS-CoV-2 pandemic which may intensify the feeling of anxiety.

Strong emotional experiences which are linked to tokophobia have an effect on the course of delivery. Research results confirmed that the duration of the first and second stage of the delivery was about one third longer. Moreover, a correlation occurred between the severity of labor-related anxiety and the frequency of emergency cesarean sections and the use of vacuum during the first labor [4]. Interestingly, Read, an English obstetrician, observed a correlation between the experienced anxiety and the occurrence of body tension which caused pain and, therefore, intensified the experience of anxiety [33,34]. The phenomenon of anxiety seems to be similar to a vicious circle in which a somatic problem is not due to pain, but due to fear of its occurrence which, in fact, intensifies the sensation of pain.

Factors influencing the severity of tokophobia included the age and financial status of the women. The present study confirmed statistical significance in terms of the young age of the respondents and the exposure to a stressor, i.e., the COVID-19 epidemic. Statistical analyses revealed that the level of education including medical education, the place of residence, professional activity and the location of delivery had no effect on the level of tokophobia. However, financial status was found to have an influence on state anxiety. Women with average financial status were characterized by higher state anxiety than women with good or very good status. Furthermore, persons with poor and very poor financial status presented high state anxiety. American research demonstrated that higher age and health-promoting behaviors during pregnancy constituted a protective factor [11]. Analogous results concerning age were obtained in the present study.

A statistically significant correlation was found between the time during the wave of the pandemic and the severity of anxiety. Both pregnancy and labor are events which significantly influence the female psyche. It is a period of elevated stress and anxiety despite the fact that it is a physiological condition [28,32]. Research on the influence of compulsory isolation on pregnant women revealed that after a month of no contact with one’s family and friends 44% of women described their mood as depressed and 14% reported such a symptom due to deteriorated financial situation and lack of work. Relationship with a partner became worse in 4%, while in 34% of women the relationship improved thanks to sharing household responsibilities. The same percentage of women reported the possibility of resting and slowing down in life. Overall, the risk of developing postpartum depression or other mental disorders is significantly increased [11]. It was confirmed by Irish research which revealed that over 50% of women were worried about their health throughout the pandemic, despite the fact that 83% of them had reported no such concerns before the pandemic. The situation of the epidemic and related restrictions intensified anxiety, fear, perception of pain which is connected with higher cesarean section rates [11]. Analogous results were obtained in the present study. According to a Polish study conducted in May 2020, individuals aged 18–24 years presented significantly higher levels of the symptoms of depression and generalized anxiety during the pandemic than the remaining age groups. It is associated with restricting one’s freedom, loneliness, lack of privacy and tiredness with the situation. However, it is not linked to the fear of losing one’s health or life which is a much more common factor triggering anxiety in a group of people older than 55 [25].

Distinguishing risk groups is the basic step in the prophylaxis of any somatic and mental disorder. Prophylaxis is predominantly based on: health education, maintenance of the continuity of medical care, establishing a rapport between the patient and midwife via appropriate communication and eliminating factors which have a negative influence on the pregnant woman. Moreover, care provided by the closest family has a positive influence on the reduction of perinatal anxiety [12]. Tokophobia is frequently underestimated by medical personnel. However, it significantly affects the course of pregnancy, labor and the health status of the mother and child postnatally. Seemingly, special advisory teams including an obstetrician, a midwife and a psychologist play an important role in the prevention and treatment of the pathological fear of labor [4]. A regulation issued by the Minister of Health which has been applicable since January 1, 2019 includes a recommendation to “assess the risk and intensity of the symptoms of depression in pregnant women” between 33 and 37 weeks of gestation. Attention was paid to prenatal education which aims at the diagnosis of psychological and emotional problems not only in women, but also in the closest family intragestationally and perinatally. Another aspect involved distinguishing mental and behavioral disorders as the risk factors of perinatal complications. Indeed, the above mentioned factors influence the prophylaxis of anxiety disorders perinatally and constitute a step towards improved psychological care of pregnant women and those in early postnatal period [12]. Polish research conducted in students during the COVID-19 pandemic showed effective forms of coping with the situation. The majority of them were motivated and diverted their attention from stressful events and a considerable number of them used rationalization, i.e., searched for the positive aspects of the situation [25].

The Earthquake Assessment Model presented by Sichel and Driscoll includes an interesting view of postpartum depression, especially when analyzed in the context of stress related to the pandemic. The mechanism of depression was described in the context of interhormonal balance, brain chemistry and stressful life events. Exposing a woman to severe long-lasting stress until labor, which is a kind of climax when anticipating a child’s birth, may lead to a situation in which the brain is no longer able to maintain balance, which results in the development of depression. The pandemic is an example of a strong stressor and it is a difficult period for the society in general. The present study demonstrated that the highest levels of anxiety was reported by the patients at the beginning of the pandemic. Pregnancy and labor constitute additional mental burden. Therefore, pregnant women are a group which is the most at risk of developing serious mental consequences in the future [11].

### 4.1. Limitations of the Study

The study was conducted in a low number of participants. Therefore, the results need to be viewed with caution. We did not identify a wide range of factors (biological, mental, and psychosocial) which may be related to anxiety and peripartum period. The second group was smaller due to the limited access to patients during the second time frame which was related to sanitary restrictions.

### 4.2. Strengths of the Study

The study was conducted in specific pandemic situation. The results indicate the directions and necessity of conducting further research in this area. The realization of the present study indirectly provided the authors with the possibility of confirming their belief that the issues tackled should not be neglected in the clinical settings.

## 5. Conclusions

The overall analysis of Labor Anxiety Questionnaire and the STAI inventory revealed a high level of anxiety in pregnant women during the SARS-CoV-2 pandemic. The majority of the respondents were characterized by a high level of situational anxiety.

Factors influencing the severity of tokophobia included the age and financial status of the women. Older women were characterized by lower levels of tokophobia than younger ones, and women with average financial status presented higher state anxiety than those with good or very good finances.

A statistically significant correlation was found between the time during the wave of the pandemic and the severity of anxiety. The respondents interviewed in October 2020 were characterized by higher levels of anxiety than those interviewed in May 2020. Furthermore, a statistically significant correlation was shown between the time during the wave of the pandemic and the level of situational anxiety.

Therefore, pregnant women or those who gave birth during the pandemic should be offered even more care and empathy in the present situation. Moreover, it seems justified to be more vigilant as regards the diagnostics of possible mental disorders in the peripartum period.

## Figures and Tables

**Table 1:** Sociodemographic data.

Data		N = Number of Subjects	%
**Marital status**	Married Domestic partnership	228 87	72.4% 27.6%
**Education**	Tertiary Secondary Vocational Primary	184 109 17 5	58.4% 34.6% 5.4% 1.6%
**Place of residence**	Village Town <10,000 inhabit. Big town >10,000<100,000 inhabit. City >100,000 inhabit. City >500,000 inhabit.	94 28 80 57 56	29.8% 8.9% 25.4% 18.1% 17.8%
**Financial status of the family**	Very good Good Average Poor	43 180 88 2	13.8% 57.1% 27.9% 0.6%
**Professional activity**	Yes No	107 208	34.0% 66%

**Table 2:** Medical history.

Data		N = Number of Subjects	%
Number of children	0 1 2 3	215 64 31 5	68.2% 20.3% 9.8% 1.6%
History of miscarriage	Yes No	55 260	17.2% 82.5%
Present pregnancy risks	Yes No	21 295	6.7% 93.7%
Regular gynecological appointments during pregnancy	Yes No	301 14	95.6% 4.4%

**Table 3:** Levels of tokophobia according to KLP questionnaire (n = 315).

Levels of Tokophobia	n	%	Chi^2^	*p*-Value
Low and moderate level of tokophobia	50	15.9%	19.387	<0.001 *
Elevated level of tokophobia	78	24.8%		
High level of tokophobia	105	33.3%		
Very high level of tokophobia	82	26.0%		

* Statistically significant p-value for the Chi^2^ test.

**Table 4:** The level of state anxiety according to STAI inventory (n = 315).

The Level of State Anxiety	n	%	Chi^2^	*p*-Value
Low levels of state anxiety	34	10.8%	82.533	<0.001 *
Moderate levels of state anxiety	117	37.1%		
High levels of state anxiety	164	52.1%		

* Statistically significant p-value for the Chi2 test.

**Table 5:** The influence of age on the level of tokophobia (n = 315).

Variable	Level of Tokophobia	Mean	F Test	*p*-Value
Age	Low and moderate level of tokophobia	29	2.952	0.033 *
	Elevated level of tokophobia	29		
	High level of tokophobia	27		
	Very high level of tokophobia	27		

F test—the Fisher–Snedecor test; * statistically significant p-value.

**Table 6:** The influence of financial status on the level state anxiety (n = 315).

Financial Status	Low		Moderate		High		Chi^2^	*p*-Value
	n	%	n	%	n	%		
Very good	13	38.2%	13	11.2%	17	10.4%	26.755	<0.001 *
Good	17	50.0%	74	63.2%	89	54.3%		
Average	4	11.8%	30	25.6%	54	32.9%		
Poor	0	0.0%	0	0.0%	2	1.2%		
Very poor	0	0.0%	0	0.0%	2	1.2%		

* Statistically significant p-value for the Chi^2^ test.

**Table 7:** The influence of the time during the wave of the pandemic and the severity of tokophobia (n = 315).

Level of Tokophobia	May		October		Chi^2^	*p*-Value
	n	%	n	%		
Low and moderate levels of tokophobia	46	17.8%	4	7.0%	8.286	0.04 *
Elevated levels of tokophobia	58	22.5%	20	35.1%		
High levels of tokophobia	90	34.9%	15	26.3%		
Very high levels of tokophobia	64	24.8%	18	31.6%		

* Statistically significant p-value for the Chi^2^ test.

**Table 8:** Correlations between the time during the wave of the pandemic and the level of situational anxiety (n = 315).

SITUATIONAL Anxiety	May		October		Chi^2^	*p*-Value
	n	%	n	%		
Low anxiety	29	11.2%	5	8.8%	18.709	<0.001 *
Moderate anxiety	109	42.2%	8	14.0%		
High anxiety	120	46.5%	44	77.2%		

* Statistically significant p-value for the Chi^2^ test.

## Data Availability

The data presented in this study are available on request from the corresponding author.
